# Increased Ghrelin but Low Ghrelin-Reactive Immunoglobulins in a Rat Model of Methotrexate Chemotherapy-Induced Anorexia

**DOI:** 10.3389/fnut.2016.00023

**Published:** 2016-07-26

**Authors:** Marie François, Kuniko Takagi, Romain Legrand, Nicolas Lucas, Stephanie Beutheu, Christine Bôle-Feysot, Aurore Cravezic, Naouel Tennoune, Jean-Claude do Rego, Moïse Coëffier, Akio Inui, Pierre Déchelotte, Sergueï O. Fetissov

**Affiliations:** ^1^Nutrition, Gut and Brain Laboratory, INSERM UMR1073, Rouen, France; ^2^Institute for Research and Innovation in Biomedicine (IRIB), Rouen University, Normandy University, Rouen, France; ^3^Animal Behavior Platform (SCAC), Rouen, France; ^4^Department of Nutrition, Rouen University Hospital, CHU Charles Nicolle, Rouen, France; ^5^Department of Psychosomatic Internal Medicine, Kagoshima University Graduate School of Medical and Dental Sciences, Kagoshima, Japan

**Keywords:** chemotherapy, intestinal inflammation, anorexia, ghrelin, autoantibodies

## Abstract

**Background and aims:**

Cancer chemotherapy is commonly accompanied by mucositis, anorexia, weight loss, and anxiety independently from cancer-induced anorexia–cachexia, further aggravating clinical outcome. Ghrelin is a peptide hormone produced in gastric mucosa that reaches the brain to stimulate appetite. In plasma, ghrelin is protected from degradation by ghrelin-reactive immunoglobulins (Ig). To analyze possible involvement of ghrelin in the chemotherapy-induced anorexia and anxiety, gastric ghrelin expression, plasma levels of ghrelin, and ghrelin-reactive IgG were studied in rats treated with methotrexate (MTX).

**Methods:**

Rats received MTX (2.5 mg/kg, subcutaneously) for three consecutive days and were killed 3 days later, at the peak of anorexia and weight loss. Control rats received phosphate-buffered saline. Preproghrelin mRNA expression in the stomach was analyzed by *in situ* hybridization. Plasma levels of ghrelin and ghrelin-reactive IgG were measured by immunoenzymatic assays and IgG affinity kinetics by surface plasmon resonance. Anxiety- and depression-like behaviors in MTX-treated anorectic and in control rats were evaluated in the elevated plus-maze and the forced-swim test, respectively.

**Results:**

In MTX-treated anorectic rats, the number of preproghrelin mRNA-producing cells was found increased (by 51.3%, *p* < 0.001) as well were plasma concentrations of both ghrelin and des-acyl-ghrelin (by 70.4%, *p* < 0.05 and 98.3%, *p* < 0.01, respectively). In contrast, plasma levels of total IgG reactive with ghrelin and des-acyl-ghrelin were drastically decreased (by 87.2 and 88.4%, respectively, both *p* < 0.001), and affinity kinetics of these IgG were characterized by increased small and big Kd, respectively. MTX-treated rats displayed increased anxiety- but not depression-like behavior.

**Conclusion:**

MTX-induced anorexia, weight loss, and anxiety are accompanied by increased ghrelin production and by a decrease of ghrelin-reactive IgG levels and affinity binding properties. Such changes of ghrelin-reactive IgG may underlie their decreased ghrelin-transporting capacities compromising ghrelin orexigenic and anxiolytic effects and contributing to chemotherapy-induced loss of appetite.

## Introduction

Cancer anorexia–cachexia syndrome is characterized by decreased food intake, weight loss, muscle tissue wasting, and psychological distress and is a major source of increased morbidity and mortality in cancer patients (1). While cancer itself may cause anorexia (2, 3), anorexia induced by chemotherapy is an independent aggravating problem, which mechanisms need to be better clarified (4). Beside anorexia, anti-cancer chemotherapy is accompanied by other side effects including intestinal mucositis, negative nitrogen balance, diarrhea, nausea and vomiting, decreasing patient’s quality of life (5–10).

Ghrelin is a 28 amino acids, peptide hormone synthesized mainly in the gastric mucosa and released in circulation in its active acylated form (11). Acylated ghrelin binds to the growth hormone secretagogue receptor (GHSR) stimulating food intake *via* a hypothalamic circuitry (12), but is unstable and rapidly degraded by plasma enzymes resulting in des-acyl ghrelin (13). Although des-acyl ghrelin is the main form of the circulating peptide, it has no orexigenic effect (14). To this day, ghrelin is the only known peripheral factor stimulating appetite, but it is also a pleiotropic hormone with multiple functions (15), including modulating mood and emotion (16). Ghrelin was indeed shown to alleviate stress-induced anxiety and may also exert antidepressive-like effects when injected to rodents (17, 18).

Considering ghrelin’s effects on appetite, mood, and emotion, it has become an important target in the research of mechanisms and treatments of several conditions of anorexia–cachexia such as in cancer anorexia and anorexia nervosa (19–21). Few studies also explored the ghrelin status in chemotherapy-induced anorexia. As such, a decrease in plasma ghrelin was reported in cisplatin-treated rats and patients (22, 23), while administration of an exogenous ghrelin stimulated food intake and minimized side effects (24). Moreover, an agonist of ghrelin improved appetite in 5-fluoruracil-treated mouse model of cancer-cachexia (25).

Methotrexate (MTX) is an anti-mitotic drug commonly used in cancer chemotherapy (26). We previously developed, in our laboratory, a rat model of MTX chemotherapy characterized by anorexia, cachexia, intestinal mucositis, impairment of absorption and digestive functions, alteration of the gut barrier, and diarrhea (27–30). While dehydration secondary to diarrhea was suggested to underlie the mechanisms of anorexia in this animal model of chemotherapy (31), possible involvement of the ghrelin system has not been studied.

In addition, we recently identified a role for ghrelin-reactive immunoglobulins (Ig) G, naturally present in plasma, in improving ghrelin’s stability by protecting it from degradation by plasma enzymes (32). Although MTX immunosuppressive properties were shown to decrease total IgG levels in rat plasma (33), its specific effect on ghrelin-reactive IgG was not studied.

Thus, in the present study, we investigated the effect of MTX on anxiety- and depression-like behavior, gastric preproghrelin mRNA-expressing cells, plasma concentrations of ghrelin as well as levels and affinity of ghrelin-reactive IgG in rats.

## Materials and Methods

### Animals

Male Sprague-Dawley rats (200–250 g) were obtained from Charles River Laboratories (France) and were housed in an air-conditioned room at 22°C with a 12:12 hours light–dark cycle (light period 07:00–19:00 hours) in a fully equipped animal facility. Rats were kept in holding cages (three rats per cage) for 1 week before the experiment, in order to acclimatize them to the housing conditions. Standard pelleted rodent chow (RM1 diet, SDS, UK) was available *ad libitum*. Three days before MTX administration, the rats were transferred to metabolism cages (Tecniplast, Lyon, France) where they were fed with the same RM1 diet but in powdered form (SDS). Drinking water was always available *ad libitum*. Animal care and experimentation were in accordance with guidelines established by the National Institutes of Health, USA and complied with both French and European Community regulations (Official Journal of the European Community L 358, 18/12/1986).

### MTX Model of Anorexia–Cachexia

Rats were divided into two groups (*n* = 12 in each) to achieve similar mean body weight: (i) MTX-treated and (ii) controls. A single dose of MTX (2.5 mg/kg, Teva Pharma, Courbevoie, France) dissolved in phosphate-buffered saline (PBS) was injected subcutaneously (S.C.) each day for three consecutive days (days 1–3). Control rats were injected S.C. with PBS. The dose and duration of MTX administration were selected based on our previous data showing typical response to MTX in rats characterized by anorexia and cachexia and loss of both lean and fat mass (31). Rats were gently handled daily during measurements of their body weight.

### Behavioral Analyses

Behavioral tests, performed to evaluate the anxious- and depressive-like behaviors in MTX-treated anorectic and in control rats, were carried out between 9.00 and 16.00 during the light period. A habituation to conditions of experimentation was performed the day preceding the experimental procedures. The rat’s spontaneous locomotion was recorded for 120 min, using a Versamax Animal Activity Monitor (AccuScan Instruments, Columbus, OH, USA). The rats were tested for their anxiety in an elevated plus-maze, which is commonly used and pharmacologically validated for anxiety testing in rodents (34). The plus-maze is elevated approximately 1 m from the floor, and the rat is given the choice of spending time in open, unprotected arms or enclosed, protected arms. The rats were tested for their depressive-like behavior in a forced-swim test. Rats were placed into a cylinder filled with tap water (25°C) to 1/3 of its volume. The total time of immobility during 15-min test procedure was registered. A rat was judged to be immobile when it floated in the water in an upright position and made only small adjustments movements to keep its head above water.

### Tissue Sampling

Rats were killed by decapitation between 10:00 and 11:00 a.m. at day 6 after beginning of MTX or PBS treatments. Trunk blood was collected into tubes containing EDTA (1 mg/ml), aprotinin (500 U/ml), and 1N HCl (1:10 vol). Plasma was separated by centrifugation at 4°C and stored at −80°C until assayed. Plasma levels of ghrelin and des-acyl ghrelin were measured using enzymatic immunoassay kits from Mitsubishi Chemical Med Corp. (Tokyo, Japan), according to manufacturer’s instructions. The manufacturer specified the sensitivity of both kits at 2.5 fmol/ml and absence of cross-reactivity between the active and des-acylated forms of ghrelin in the corresponding detection assays. For *in situ* hybridization, the stomach tissue was frozen on dry ice, stored at −80°C, then cut in a cryostat (Leica Microsystems, Nanterre, France) to obtain 14-μm thick transversal sections collected on Superfrost glass slides (Thermo Scientific, Braunschweig, Germany).

### *In Situ* Hybridization

All solutions were made using diethylpyrocarbonate (DEPC, Sigma, St. Louis, MO, USA) treated water. Stomach sections were fixed with 4% paraformaldehyde in PBS, pH 7.5. After washing with PBS for 5 min, the sections were incubated with 0.5M HCl in DEPC water for 5 min and then washed in PBS twice for 3 min. Further, the sections were treated with 0.25% acetic anhydride in 0.1M triethanolamine, pH 8.0, for 20 min. The sections were washed in PBS twice for 3 min, and immersed in a graded ethanol series (70, 80, and 99.5%) for 2 min each. Finally, sections were dried for 30 min and then stored at −80°C.

RNA probes specific to preproghrelin mRNA (Accession number: NM_021669.2, National Center for Biotechnology Information, Bethesda, MD, USA) were prepared from rat stomach mRNA. The rat stomach extracted mRNA was reverse-transcribed to generate cDNA using the iScript select cDNA synthesis kit (Bio-Rad, Hercules, CA, USA). The cDNA was amplified using the following specific primers: ghrelin-F: 5′-AGCACCAGAAAGCCCAGCAGAGAA-3′ and ghrelin-R: 5′-TTGCAGAGGAGGCAGAAGCTGGAT-3′ (product size of 335 bp from position 121–455) (Invitrogen, Carlsbad, CA, USA). The PCR fragment was gel purified using QIAquick Gel extraction kit (Qiagen) and subcloned into PCR1II-TOPO vector (Invitrogen, Carlsbad, CA, USA). The sequence of cDNA probe was confirmed by nucleotide sequencing (KIGene, Stockholm, Sweden). The plasmids were linearized using restriction enzyme BAmHI and *Xba*I (Promega, Madison, WI, USA) and transcribed to generate sense and antisense RNA probes. *In vitro* transcription and labeling were carried out using SP6/T7 RNA polymerases (Ambion, Austin, TX, USA) and digoxigenin (DIG) RNA labeling mix (Roche Diagnostics, Mannheim, Germany) according to the manufacturer’s instructions. The transcripts were purified using NucAway Spin Columns (Ambion). Sense probes were used as negative controls.

Sections were prehybridized in a humidified chamber using 50% (*v/v*) deionized formamide, 0.1M Tris–HCl (pH 7.6), 50 mM EDTA (pH 8.0), 40 mM NaCl, 1.25 mg/ml yeast tRNA (Roche), and 5× Denhardt’s solution for 4–6 h at 55°C followed by hybridization overnight (14–16 h) at 55°C. The labeled probes were diluted to a final concentration 0.8 ng/μl in solution containing 50% (*v/v*) deionized formamide, 0.3M NaCl, 0.1 M DTT, 10% (vol/vol) dextran sulfate, and 1× Grundmix solution. Grundmix solution is made of 0.2M Tris–HCl (pH 7.6), 5 mg/ml yeast tRNA (Roche), 1 mg/ml poly-A-RNA (Roche), 10× Denhardt’s solution, and 10 mM EDTA (pH 8.0). After hybridization, sections were washed with constant stirring as follows: twice for 30 min in 1× SSC with 0.1% SDS at 55°C, 1 h in 50% (*v/v*) formamide/0.5× SSC at 55°C, 5 min in 1× SSC with 0.1% SDS at 55°C, 30 min in 36 μg/ml RNase A diluted in RNase A buffer at 37°C, and twice for 10 min in 1× SSC with 0.1% SDS at 55°C. RNase A buffer contains 0.5M NaCl, 10 mM Tris (pH 8.0), and 0.5 mM EDTA. The sections were then incubated three times for 5 min in buffer 1 (100 mM Maleic acid, pH 7.5, 150 mM NaCl, 0.02% tween 20), immersed in 1% blocking reagent (Roche) diluted in buffer 1 for 20 min, and incubated with the alkaline phosphatase-conjugated anti-DIG antibody (Roche) diluted at 1:200 in buffer 1 with 1% blocking reagent at 4°C overnight. The sections were then washed in buffer 1 for 5 min three times, in buffer 2 (100 mM Tris, pH 9.5, 100 mM NaCl, 0.05% tween 20) for 5 min, and in buffer 2 with 5 mM of tetramisole hydrochloride (Sigma) for 5 min. A chromagen solution made of 337 g/ml nitro-blue tetrazolium chloride (NBT) and 175 g/ml 5-bromo-4-chloro-3′-indolyphosphate p-toluidine salt (BCIP) (Roche) in buffer 2 with 5 mM of tetramisole hydrochloride (Sigma) was made, and the sections were incubated for 24 h. The reaction was stopped with PBS. The sections were then washed with distilled water, mounted in a solution of PBS and glycerol (85%), and viewed under a light microscope (Axioskop, Zeiss, Germany). Pictures from the *in situ* hybridization were taken with an objective 20×. Total number of preproghrelin mRNA-positive cells was counted using Image J 1.45s software (National Institutes of Health, Bethesda, MD, USA) from five representative sections of each mouse. The square area including the gastric mucosa and sub-mucosa were delimited in Image J 1.45s in each picture, and the number of positive cells in the measured surface area was calculated.

### Ghrelin-Reactive Immunoglobulins

Ghrelin-reactive Ig levels were measured in rat plasma using an enzyme-linked immunosorbent assay (ELISA) accordingly to a published protocol (35). Briefly, 96-well plates (Nunc Immunoplate, Rochester, NY, USA) were coated with 2 mg/ml of rat ghrelin (Peptide institute, Inc., Japan) in 0.5M sodium carbonate (Na_2_CO_3_), 0.5M sodium bicarbonate (NaHCO_3_), pH 9.6, and incubated for 72 h at 4°C. Plates were washed using PBS (0.05% Tween20, pH 7.4). Plasma was diluted 1:100 in either normal buffer (PBS, 0.02% sodium azide, pH 7.4) to measure free IgG or in dissociative 3M NaCl, 1.5M glycine (pH 8.9) buffer, to measure total ghrelin IgG levels. After incubation at 4°C overnight, plates were washed and incubated for 3 h with alkaline phosphatase-conjugated secondary antibody (anti-rat IgG; Jackson ImmunoResearch, West Grove, PA, USA). The reaction was developed for 40 min with 150 μl *p*-nitrophenyl phosphatase substrate solution (Sigma, St Louis, MO, USA) at 1 mg/ml in 0.1M Tris–HCl, 0.1M NaCl, 5 mM magnesium chloride hexahydrate (MgCl_2_⋅6H_2_0), pH 9.5. About 100 μl sodium hydroxyde (NaOH) (2M) was added to stop the reaction and optical density (OD) was measured at 405 nm in an ELISA plate reader.

### IgG Purification

Plasma samples were purified using a Melon Gel kit according to the manufacturer instruction (Thermo Fisher Scientific, Rockford, IL, USA). After the purification, IgG was frozen at −30°C and lyophilized overnight. Then, they were suspended in the HBS-EP buffer (GE Healthcare). The IgG concentration of each sample was determined with a Nanodrop^®^, and HBS-EP buffer was used as a blank.

### Affinity Kinetics Analysis

Affinity kinetics of mice IgG for ghrelin and des-acyl-ghrelin was determined by surface plasmon resonance (SPR) on a BIAcore 1000 instrument (GE Healthcare) according to a published protocol (36). In brief, rat ghrelin (Peptide institute) was diluted at 0.5 mg/ml in 10 mM sodium acetate buffer (pH 5.0) (GE Healthcare) and was covalently coupled on the sensor chip CM5 (GE Healthcare), using the amine coupling kit (GE Healthcare). All the measurements were done on the same sensor chip. For the affinity kinetic analysis, a multi-cycle method was run with five serial dilutions at 1/2 of each IgG sample: 1680–105 nM plus a duplicate of a blank (buffer only) at the beginning and at the end of the kinetic. During each cycle, the flow speed was 30 μl/min. About 60 μl of each sample dilution were injected correspond to 2-min period followed by 5 min of dissociation. Then, between injections of each dilution, the binding surface was regenerated with 10 mM NaOH resulting in the return of the sensorgram to the same baseline. The affinity kinetic data were analysed using BiaEvaluation 4.1.1 programme (GE Healthcare). For fitting kinetic data, the Langmuir’s 1:1 model was used, and the sample values were corrected by subtracting the blank values resulting from the injection of HBS-EP buffer.

### Statistical Analysis

Group differences were statistically analyzed using GraphPad Prism 5.02 (GraphPad software Inc., San Diego, CA, USA). Differences in daily food intake, body weight, and water intake were analyzed by a two-way repeated measurements (RM) ANOVA taking in account effects of time and treatment followed by the Bonferroni post-tests. Individual groups were compared using the Student’s *t*-test or Mann–Whitney test according to the Kolmogorov–Smirnov (KS) normality test, and *p* < 0.05 was considered as significant.

## Results

### Food Intake, Body Weight, and Water Intake

**Figure 1:**
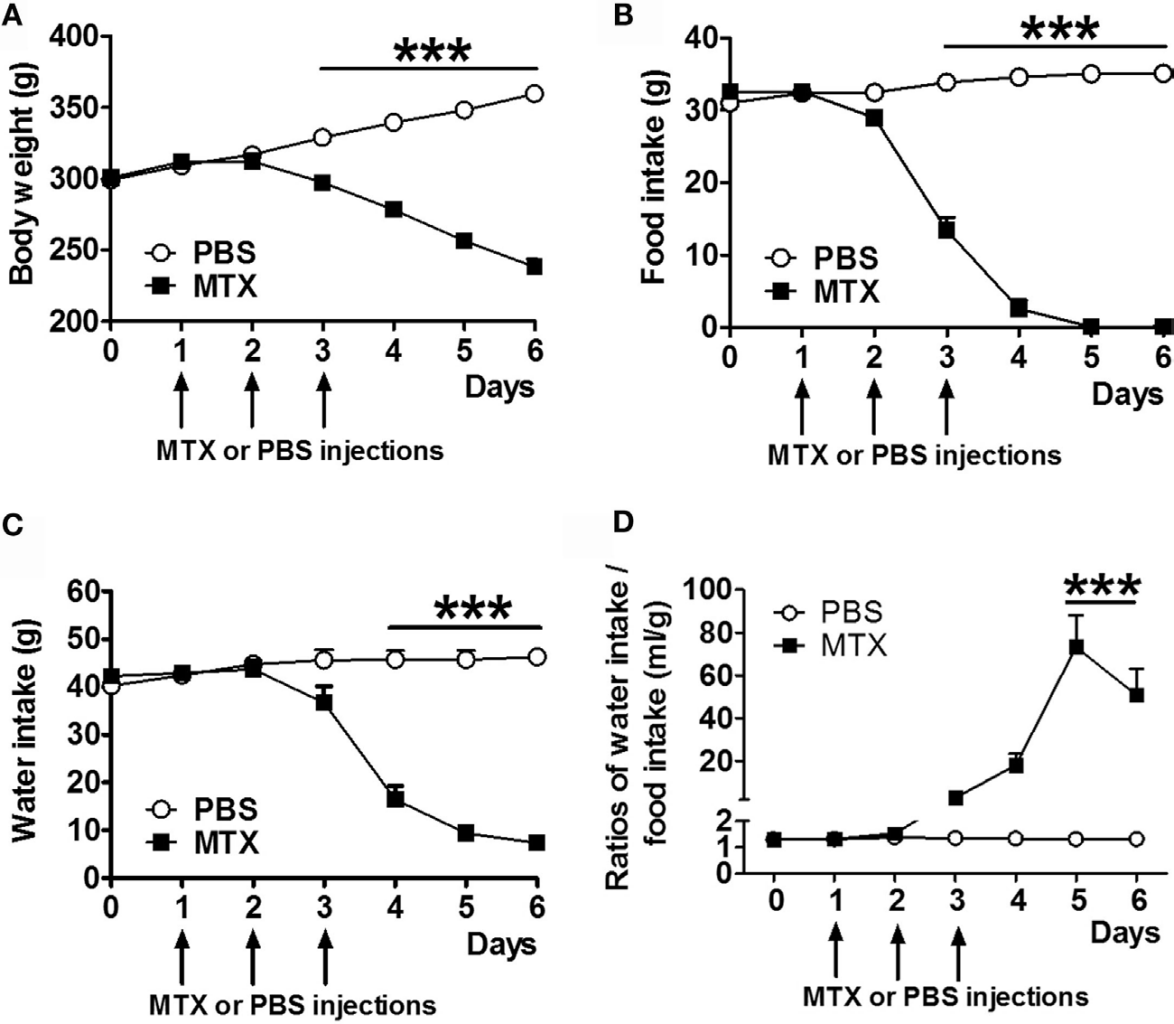
**Body weight (A), daily food intake (B), and water intake (C) of MTX-treated and PBS-treated control rats (both, *n* = 10)**. Ratios of daily water to food intakes **(D)**. Two-way RM ANOVA with Bonferroni post-tests ***p < 0.001, effects of time *p* < 0.0001 (mean ± SEM).

Methotrexate-treated rats displayed significant body weight loss (Figure 1A) and decreased food intake (Figure 1B) starting from day 3 after the first MTX injection. Water intake was reduced from day 4 (Figure 1C). Food intake was completely inhibited in MTX-treated rats at days 5 and 6 when they lost 33.7% of body weight as compared to control rats. The ratios of water to food intake were increased in anorectic MTX-treated rats (Figure 1D).

### Preproghrelin mRNA-Producing Cells

**Figure 2:**
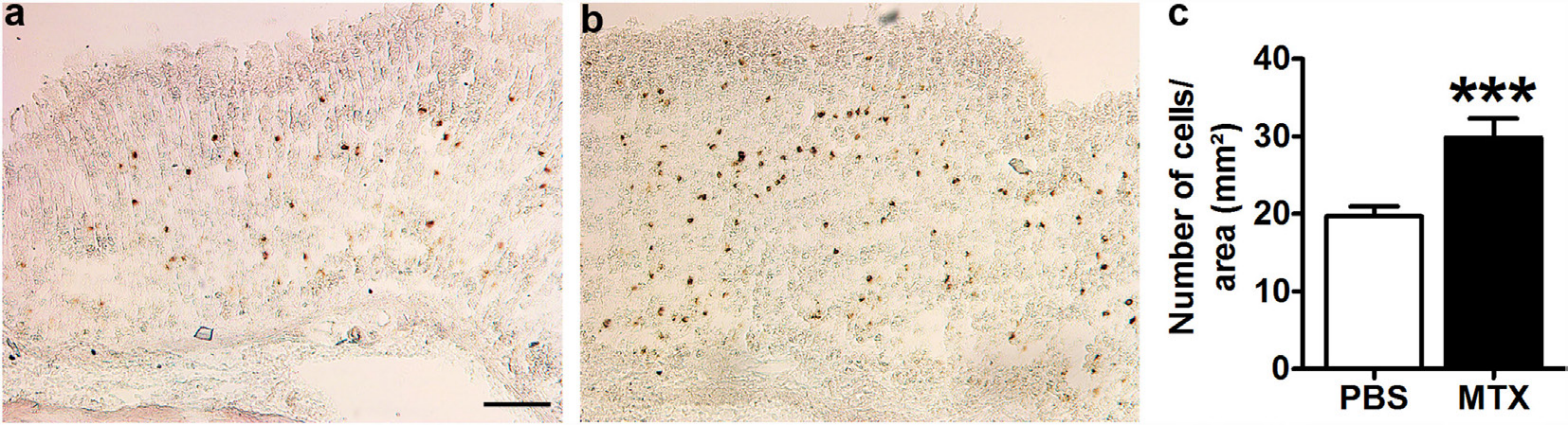
**Representative microphotographs of preproghrelin mRNA-expressing cells in the stomach of PBS-treated control rats (*n* = 10) (A) and MTX-treated rats (*n* = 7) (B) revealed by *in situ* hybridization with DIG-labeled preproghrelin anti-sense riboprobes**. **(C)** Quantification of the number of preproghrelin mRNA-positive cells in the gastric mucosal and submucosal layers of control and MTX-treated rats. Student’s *t*-test, ***p < 0.001, KS test *p* > 0.1 both (mean ± SEM). Scale bar, 100 μm.

*In situ* hybridization revealed the presence of preproghrelin mRNA-expressing cells in the mucosal and sub-mucosal layers of the gastric wall of all control and MTX-treated rats (Figures 2A,B). The negative control did not show any positive cells (not shown). The number of preproghrelin mRNA-producing cells was increased by 51.3% in MTX-treated rats as compared to controls (Figure 2C). The gastric mucosa in MTX-treated rats did not show signs of atrophy.

### Plasma Levels of Ghrelin

**Figure 3:**
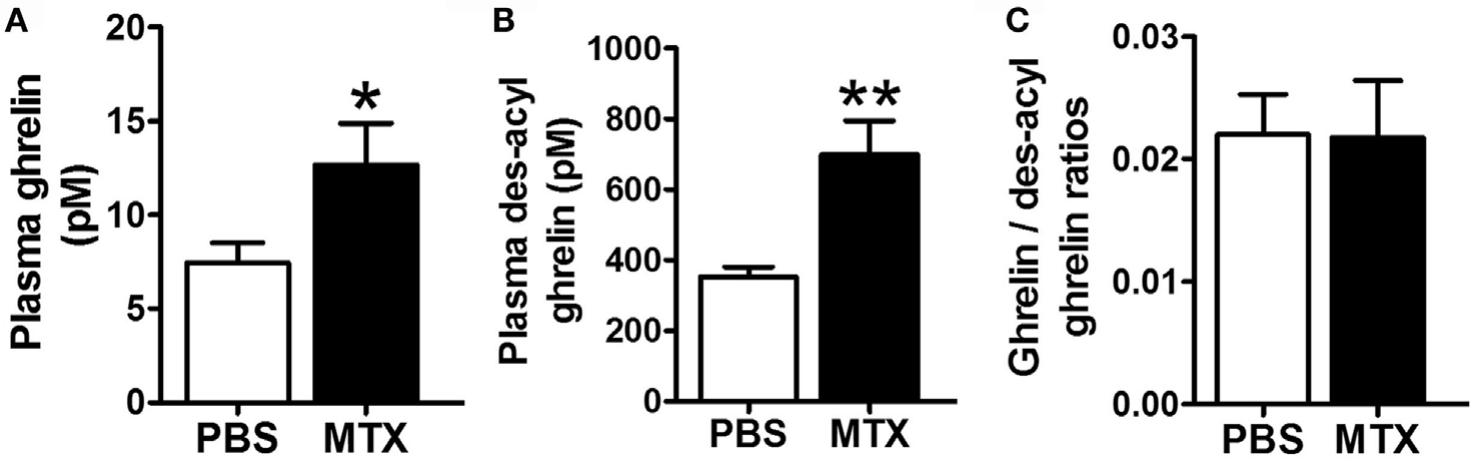
**Plasma concentrations of ghrelin (A), des-acyl ghrelin (B), and their ratios (C) in PBS-treated controls (*n* = 8) and MTX-treated (*n* = 10)**. *p < 0.05, Mann–Whitney test, KS test *p* = 0.03 and *p* > 0.1 and **p < 0.01 Student’s *t*-test, KS test *p* > 0.1 both (mean ± SEM).

Plasma levels of ghrelin and des-acyl ghrelin were robustly increased in MTX-treated vs. control rats (Figures 3A,B) while their ratios remained similar (Figure 3C).

### Ghrelin-Reactive IgG

**Figure 4:**
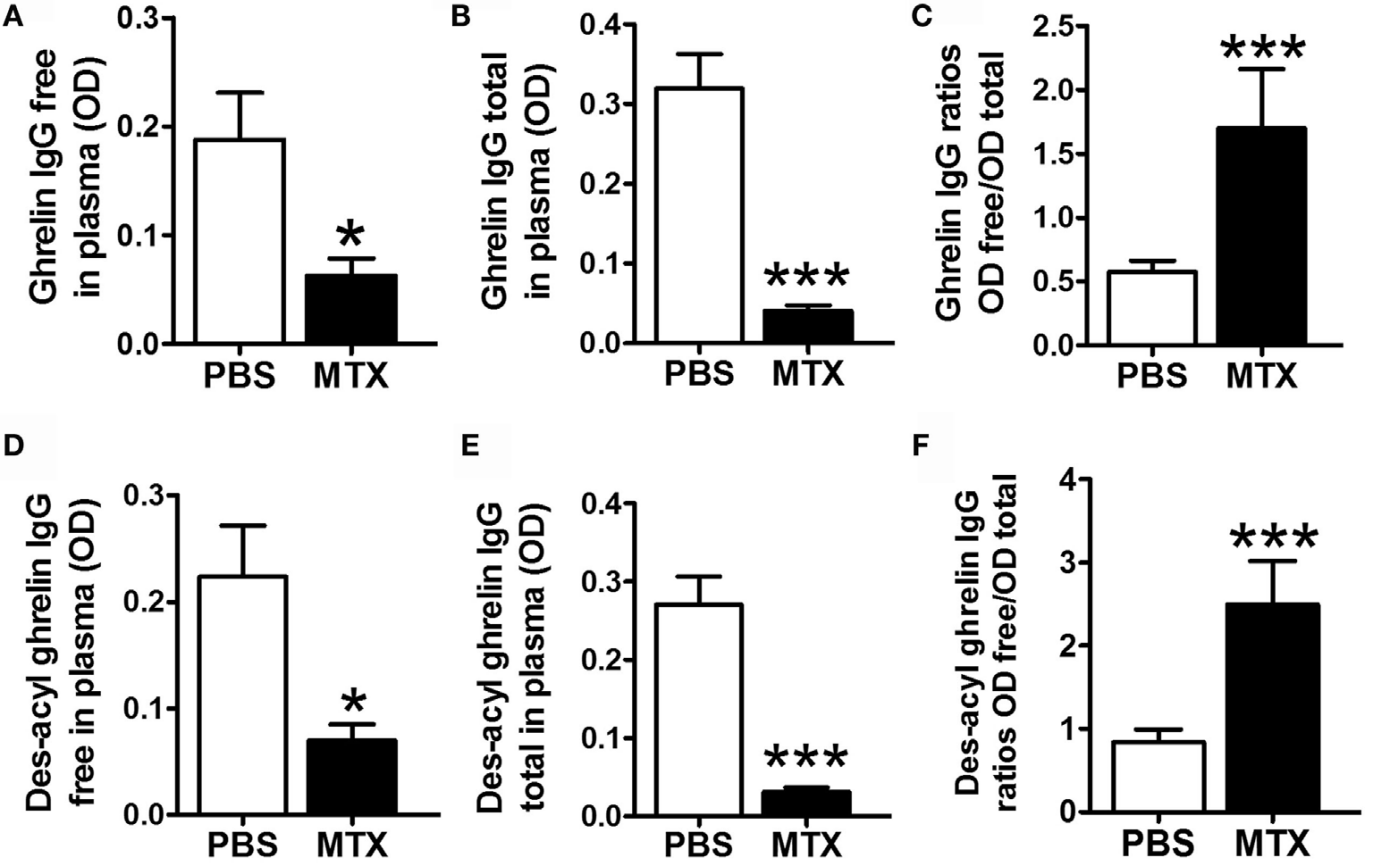
**Plasma concentrations of free (A,D) and total (B,E) IgG reactive with ghrelin (A,B) and des-acyl ghrelin (D,E) in MTX-treated (*n* = 10) and PBS-treated control rats (*n* = 10)**. Mann–Whitney or Student’s *t*-test **(A,E)** according to the KS normality tests, *p < 0.05, **p < 0.01, ***p < 0.001. KS tests, **(A)** *p* > 0.1 and *p* = 0.08, **(B)** *p* > 0.1 and *p* = 0.03, **(C)** *p* > 0.1 and *p* = 0.003, **(D)** *p* > 0.1 and *p* = 0.005, **(E)** *p* > 0.1 and *p* = 0.1, **(F)** *p* > 0.1 and *p* = 0.006 (mean ± SEM).

Methotrexate-treated rats showed decreased plasma levels of both free and total acyl ghrelin-reactive IgG (Figures 4A,B) and free and total des-acyl ghrelin-reactive IgG (Figures 4D,E). The ratios of free to total IgG reactive with both ghrelin and des-acyl-ghrelin were increased in MTX-treated rats (Figures 4C,F).

### Affinity Kinetics of IgG for Ghrelin

**Figure 5:**
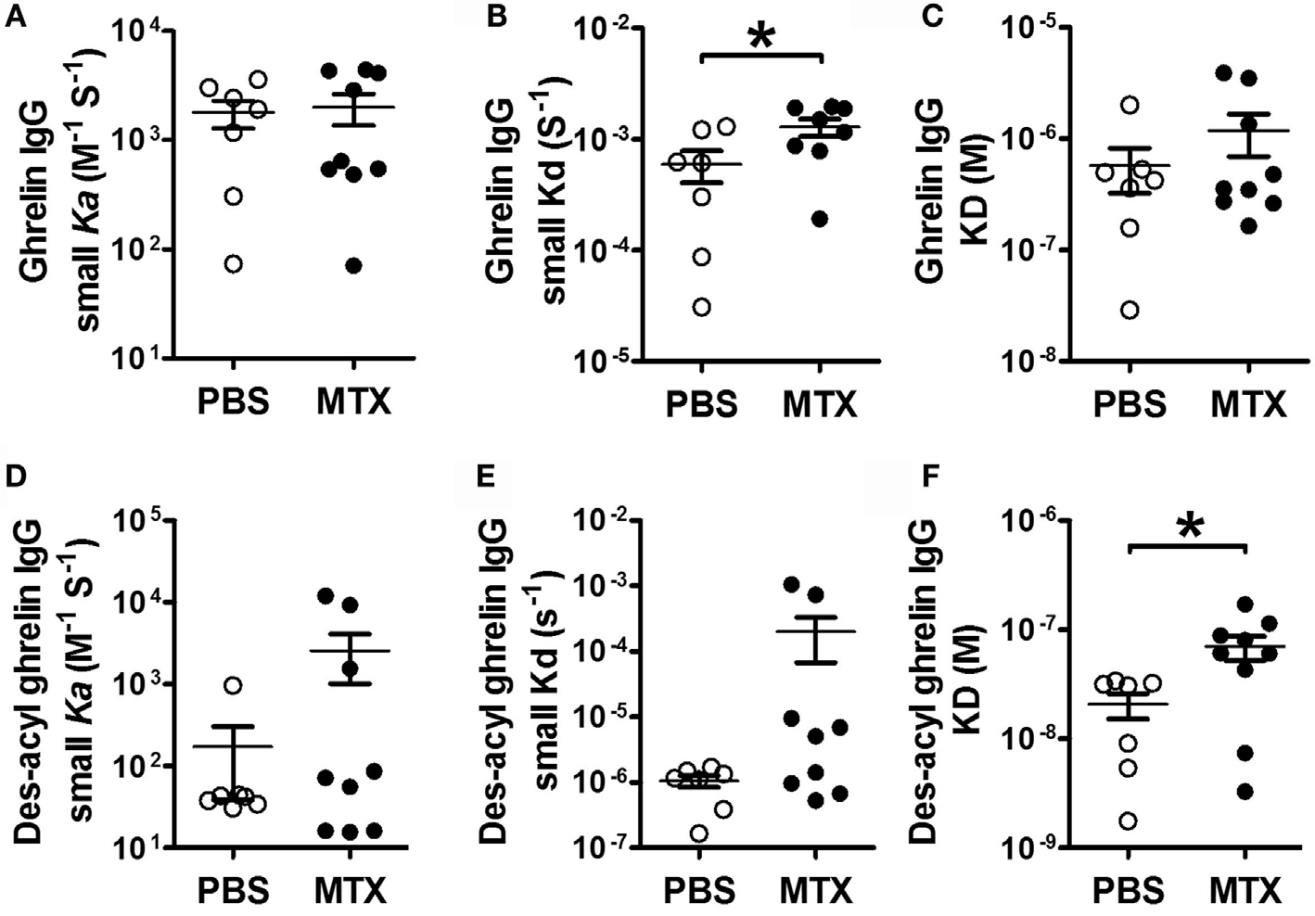
**SPR affinity kinetics analysis of ghrelin- (A–C) and des-acyl ghrelin (D–F)-reactive IgG in MTX-treated (*n* = 9) and PBS-treated control rats (*n* = 7)**. The affinity kinetic parameters are: the association rates (small ka) **(A,D)**, the dissociation rates (small kd) **(B,E)** and the dissociation equilibrium constants (KD) **(C,F)**. Student’s *t*-test **(B)** or Mann–Whitney test according to the KS normality tests, *p < 0.05. KS tests, **(B)**, *p* > 0.1 both and (**F)**, *p* = 0.02 and *p* > 0.1 (mean ± SEM).

The analysis of affinity kinetics using SPR revealed increased dissociation rates (small Kd) of IgG for ghrelin in MTX-treated rats (Figure 5B), who also displayed increased dissociation equilibrium constants (big KD) of their IgG for des-acyl-ghrelin (Figure 5F). Other parameters of affinity kinetics were not significantly different among the groups (Figures 5A,C,D,E).

### Anxious- and Depressive-Like Behavior

Locomotor activity, including time of movement and crossed distance, was unchanged between MTX and control groups (Table 1). The number of entries in both open and closed arms in the elevated plus-maze was decreased in the MTX-treated rats (Table 1). The time spent in both open and closed areas was not significantly different between the groups, although MTX-treated rats tended to spend less time in open arms. There was no either significant differences between MTX and control groups in the immobility time of the forced-swim test (Table 1).

**Table 1:** **Behavioral tests results**.

		PBS-controls (mean ± SEM)	MTX-treated (mean ± SEM)	Statistical test	*p-*values
Locomotor activity	Time (s)	466.0 ± 125.0	449.2 ± 138.4	Student’s *t*-test	0.46
	Distance (cm)	5218 ± 1484	4661 ± 1519	Student’s *t*-test	0.4
Forced-swim test	Immobility time (s)	3.2 ± 0.15	3.23 ± 0.09	Student’s *t*-test	0.43
Elevated plus-maze	Total distance (cm)	1486 ± 114.7	1728 ± 209.3	Mann–Whitney	0.14
Closed arms	Distance (cm)	1273 ± 88.08	1498 ± 194.7	Student’s *t*-test	0.13
	Entries	23.38 ± 3.21	15.50 ± 2.922	Mann–Whitney	**0.016**
	Time (s)	198.6 ± 8.57	212.9 ± 12.37	Student’s *t*-test	0.18
Open arms + central area	Distance (cm)	213.0 ± 24.30	230.0 ± 63.59	Student’s *t*-test	0.39
	Entries	31.88 ± 2.95	18.00 ± 3.31	Mann–Whitney	**0.007**
	Time (s)	98.17 ± 8.84	73.11 ± 12.78	Mann–Whitney	0.05

*Results of the locomotor activity test (time and distance), the forced-swim test (immobility time), and the elevated plus-maze test (distance, entries and time spent in closed or open arms) in PBS-treated controls (*n* = 8) and MTX-treated rats (*n* = 8). Student’s or Mann–Whitney tests were used according the Kolmogorov–Smirnov normality test, and *p* < 0.05 was considered as significant (boldface values)*.

## Discussion

In this study, we analyzed the effects of MTX on preproghrelin mRNA-expressing cells and plasma levels of ghrelin and ghrelin-reactive IgG in a rat model of chemotherapy-induced anorexia. We found that the number of preproghrelin mRNA-producing cells was increased in rats treated with MTX, accompanied by increased plasma concentrations of both ghrelin and des-acyl-ghrelin. In contrast, plasma levels of ghrelin- and des-acyl-ghrelin-reactive IgG were strongly decreased in MTX-treated anorectic rats and further displayed altered properties of their affinity kinetics, indicative of reduced biding of ghrelin and des-acyl-ghrelin. To our knowledge, this is the first report on the effect of MTX on the ghrelinergic system. Moreover, we showed that MTX-treated rats displayed increased anxiety- but not depression-like behavior.

First of all, our data revealed that MTX-induced anorexia is not due to a decreased ghrelin production by the stomach, which could be expected because of MTX-induced mucositis. In contrast, we found that the number of preproghrelin mRNA-expressing cells was increased in MTX-treated rats supporting the preservation of a normal feedback control from the negative energy balance that upregulates ghrelin’s gene expression (37). Our previous work using the same rat model of MTX chemotherapy supported a key role of dehydration in appearance of anorexia and weight loss (31). The underlying pathophysiological process included intestinal mucositis-induced diarrhea, fluids loss, and activation of brain structures signaling dehydration including oxytocin neurons in the hypothalamic paraventricular nucleus (31). Such oxytocin neurons display important anorexigenic activity counteracting the ghrelin-activated brain hunger pathway (38, 39). Increased water to food intake ratios during anorexia in MTX-treated rats suggest that decreased food intake was not secondary to inhibited drinking. Although the ghrelin production does not appear to cause anorexia in the MTX rat model, its altered signaling may still contribute to the insufficient orexigenic effects of ghrelin to reduce the negative energy balance and to counteract other side effects of chemotherapy.

Negative energy balance is, indeed, typically accompanied by upregulation of the ghrelin gene expression and its plasma levels. For instance, patients with anorexia nervosa display elevated ghrelin concentrations, associated with lower body mass index and fat mass (40–43). Chronic food restriction in rodents is also accompanied by increased plasma ghrelin (44–47). Moreover, we have recently showed that the number of preproghrelin mRNA-expressing cells in the stomach of mice with activity-based anorexia and chronic food restriction was increased proportionally to their body weight loss (48). Thus, the present data of the increased number of ghrelin-expressing cells and ghrelin levels in plasma after MTX treatment are in agreement with the results in both humans and rodents characterized by negative energy balance. However, our results are contrasting with a study showing an impaired ghrelin production in cancer chemotherapy-induced anorexia using cisplatin (22, 23). It suggests that ghrelin system might be differentially affected by different types of chemotherapy, which may involve impaired secretion by the gastric A/X-like-type cells, decreased production/activity of the ghrelin *O*-acyltransferase, enzyme responsible for the posttranscriptional acylation of ghrelin precursor (49), or an increased degradation of total and acylated ghrelin by plasmatic enzymes such as lysophospholipase 1 (50).

Considering the immunosuppressive properties of MTX, we tested if MTX alters production of ghrelin-reactive IgG. Both free and total levels of IgG reacting with either acylated or des-acylated forms of ghrelin were strongly decreased under the effect of MTX. An impairment of ghrelin-reactive IgG production can be due to MTX-induced mucositis affecting gut lymphoid tissue and other lymphoid organs but is not likely to be a result of food restriction *per se* since we previously showed that total plasma IgG concentrations were increased in rats pair-fed with rats displaying MTX-induced anorexia (33). Ghrelin-reactive IgG protect ghrelin from degradation and enhance its orexigenic effect depending on IgG affinity for ghrelin, which is slightly increased in obese humans, mice, and rats (32, 51). It suggests that ghrelin can be transported by plasmatic IgG toward its receptors and that increased, but still remaining in the micromolar range, affinity of IgG is associated with its better transportation. Inversely, a decrease in affinity of IgG for ghrelin may reduce such transportation ability. Therefore, low levels of ghrelin-reactive IgG and changes of their affinity kinetics showing increased dissociation of ghrelin, may diminish ghrelin’s stability and signaling. Increased ratios of free to total ghrelin-reactive IgG may also reflect their decreased ability to form immune complexes with ghrelin. Similarly to such changes in MTX anorectic rats, previous studies in patients with anorexia nervosa also found low plasma levels of ghrelin-reactive IgG, which were increased after refeeding (52) and were also characterized by increased dissociation rates (32). Further experiments will be required to test if administration of ghrelin-reactive IgG may enhance ghrelin’s orexigenic effects to counteract anorexia, e.g., during cancer chemotherapy. Although our recent study using such experimental approach in mice with activity-based anorexia did not show superior results as compared to the use of ghrelin alone, a short-time access to food in this animal model of anorexia was a limitation to fully evaluate a therapeutic potential of increasing ghrelin’s stability using IgG (47).

Distress, anxiety, and depression are often observed in cancer patients (53) and can be aggravated by chemotherapy (54, 55), highlighting high psychological and psychiatric co-morbidity. Although the mechanisms of these diseases are not fully understood, there is growing evidence of the role of the gut–brain axis, including gut microbiota-derived signals acting directly or *via* inflammatory mediators on the nervous system (56–58). Cancer chemotherapy, primary aimed at inhibiting tumor cell proliferation, also destroys gut mucosal layer composed of continuously renewed cells, resulting in increased gut barrier permeability such as in our MTX chemotherapy model (28). It is hence likely that MTX-induced damage of the gut barrier may be accompanied by increased anxiety- and depression-like behavior in rats. In this study, we found that MTX did not induce a depressive-like behavior in rats in the forced-swim test, but decreased the number of entries in both open and closed arms in the elevated plus-maze. Considering their normal locomotor pre-test activity, these changes indicate that MTX may increase anxiety distinctly from the despair-related depressive-like symptoms of the forced-swim test. Because ghrelin plays a role in the stress-induced emotional regulation (16), the impairment of ghrelin signaling could also contribute to the elevated anxiety in MTX-treated rats. Changes of ghrelin-reactive IgG, which were inhibited by MTX, may potentially contribute to the increased anxiety. In fact, we have recently found that serum levels of ghrelin-reactive IgM and IgG correlate with anxiety and the stress-response in a general population of adolescents (59). Although these associations were weak and probably reflected some physiological variations of anxiety in healthy adolescents with normal levels of ghrelin-reactive autoantibodies, a dramatic decrease of ghrelin-reactive IgG in MTX-treated rats may potentially diminish ghrelin’s protective role in stress-induced anxiety.

## Conclusion

In conclusion, this study reveals that MTX chemotherapy-induced anorexia–cachexia in rats is accompanied by increased ghrelin production but lower levels and binding affinity kinetics of IgG that transport ghrelin and protect it from degradation. Such changes of ghrelin-transporting IgG may compromise ghrelin signaling potentially contributing to anorexia, weight loss and anxiety in MTX chemotherapy-induced anorexia–cachexia.

## Author Contributions

SF designed the study and together with MF wrote the manuscript. MF performed ISH and behavioral experiments and analyzed the data. RL and NL analyzed plasma ghrelin and IgG. KT, SB, CB-F, AC, and NT participated in the experiments. J-CdR, MC, AI, and PD contributed to the study design. All authors contributed to and have approved the final manuscript.

## Conflict of Interest Statement

SF and PD are co-founders of TargEDys SA and PD received research grants from Nestlé, Fresenius Kabi and honoraria for speeches and consulting from Nestlé, Fresenius-Kabi and Aguettant, other authors declared that they have no conflict of interest.
